# Quantum-Inspired Adaptive Meta-Heuristic–Machine Learning framework for resilient and energy-efficient task scheduling in multi-cloud ecosystems

**DOI:** 10.1038/s41598-026-43125-3

**Published:** 2026-03-12

**Authors:** Nomula Divya, M. N. V. Kiranbabu, G. Charles Babu

**Affiliations:** 1 Department of Computer Science and Engineering, Koneru Lakshmaiah Education Foundation, Vaddeswaram, Guntur, Andhra Pradesh India; 2 Department of Computer Science and Engineering, Gokaraju Rangaraju Institute of Engineering and Technology, Hyderabad, Telangana India

**Keywords:** Quantum-inspired optimization, Whale optimization algorithm, Federated learning, Multi-cloud scheduling, Energy efficiency, Resilience, Meta-heuristics, Green cloud computing, Task placement, Fault tolerance, Engineering, Mathematics and computing

## Abstract

With the exponential growth of multi-cloud, spearheaded by latency-sensitive workloads, edge inclusion and heterogeneous resource pools; there is a dire need for scheduling methods which are energy neutral as well as robust against dynamic operational stress. In this work, a new bi-directional optimization–prediction system for intelligent task scheduling over federated cloud environments called Quantum-Inspired Adaptive Meta-Heuristic–Machine Learning (QI-AMHML) is proposed. Central to this is the Reinforced Quantum Whale Optimization Algorithm (RQWOA) which immerses quantum probability amplitudes and wavefunction-motivated search dynamics into classical whale optimization. Together with a federated gradient boosting scheduler, the framework establishes a continuous co-evolution between search and predictive guidance that allows proactive placement decisions under changing load and fault scenarios. The method combines resilience, energy models and load balancing constraints into a single multi-objective optimization. In evaluation based on a hybrid dataset, called MultiCloudSynth-2025, created by merging real-world traces from Google Cloud, Microsoft Azure, and Alibaba Cloud with synthetic burst (load) and fault events; QI-AMHML decreased energy consumption up to 38.2%, increased an average service delay by 33.5% and in clustered multi-fault scenarios maintained high resilience scores. We validated these results under large-scale simulations and live shadow deployments, showing that quantum-inspired search reinforced by federated predictive models can provide sustainable fault-tolerant schedule performance in real-world modern multiclustered hybrid clouds.

## Introduction

In the past decade, a cloud-oriented expansion toward multi-cloud has transmuted frommere resource-pooling to hyper-distributed cross-platform orchestration serving billions of latency-sensitive tasks every day. The task scheduling problem has become a fundamental issue in multi-cloud efficiency, given the on-the-fly nature of IoT-driven workloads, edge AI inference and latency-critical 5G applications pulsing through new multi-cloud environments. This research have seen many times in my work how small improvements to scheduling efficiency can have direct cascading reductions in energy consumption, operational cost and system downtime, however achieving those gains where there is significant variability within the workload is far from trivial.

Classic scheduling algorithms — either deterministic (for example, earliest-deadline first, min-min heuristics) or meta-heuristic (for instance, particle swarm optimization etc.) start to suffer when it comes to high-variance and highly volatile load scenarios. These methods tend to either converge slowly in adapting to changing conditions or over-fit to prevailing workload distributions making them brittle in the presence of unexpected workload anomalies. Similarly, even off-the-shelf machine learning (ML) schedulers that are pure and trained offline often struggle to perform satisfactorily in concept drift situations, when the incoming task distribution evolves faster than retraining cycles can keep up with.

To address this challenge, in this paper we put forward a Quantum-inspired Adaptive Meta-heuristic – Machine Learning (QI-AMHML) framework. This research start with a Reinforced Quantum Whale Optimization Algorithm (RQWOA), inspired by quantum search, to break local minima and align learning with an ensemble ML model (federated Boosting classifier) across many clouds without moving sensitive task data out. Through embedding the search wavefunctions inspired by quantum mechanics directly into the meta-heuristic update rules, this research show that algorithm was able to better balance between exploration and exploitation under multi-cloud heterogeneity.

The novelty comprises not only the merge of meta-heuristics and ML (a concept in hybrid schedulers), but also a reinforced dual feedback loop between those two: The meta-heuristic search space is being shaped by predictive ML feedback on a per scheduling iteration basis, while the ML model is re-weighted according to quantum-inflected search trajectories. And as this research will demonstrate, this co-evolutionary loop results in a schedular that is not only adaptive in the short term but stable across sustained dynamic workload changes.

Mathematically, the task scheduling objective can be expressed as a multi-objective optimization problem, where we simultaneously minimize energy consumption, ${E}_{total}$, and average service delay, ${D}_{avg}$, while maximizing throughput, ${T}_{through}$. The composite objective function can be formalized as:

1 $$min _{S} F\left( S \right) = \alpha \cdot \left( {\frac{{E_{{total\left( S \right)}} }}{{E_{{\max }} }}} \right) + \beta \cdot \left( {\frac{{D_{{avg\left( S \right)}} }}{{D_{{\max }} }}} \right) - \gamma \cdot \left( {\frac{{T_{{through\left( S \right)}} }}{{T_{{\max }} }}} \right)$$

where $F\left(S\right)$ denotes the scheduling strategy, and ($\alpha,\beta,\gamma$) are dynamically tuned weights.

In my experiments, these weights were not fixed; instead, they evolved as functions of cloud load entropy, ${H}_{load\left(t\right)}$, which this research measured as:

2 $$H_{{load\left( t \right)}} = - \sum _{{i = 1}}^{M} p_{{i\left( t \right)}} \cdot \log ^{2} p_{{i\left( t \right)}}$$

where ${p}_{i\left(t\right)}$ is the proportion of workload on cloud $i$ at time $t$, and $M$ is the total number of clouds in the federation.

The RQWOA introduces a quantum probability amplitude into the whale position update mechanism. In classical Whale Optimization, the position ${x}_{i}^{}$ is updated via encircling prey or spiral movement. In my quantum variant, each position update is governed by a quantum superposition state:

3 $$x_{i}^{{t + 1}} = \psi \cdot x_{{best}}^{t} + \left( {1 - \psi } \right) \cdot x_{i}^{t} + \xi \cdot \varphi \left( \theta \right)$$

where $\psi$ is a probabilistic amplitude derived from a quantum wavefunction, $\xi$ is a scaling parameter, and $\varPhi\left(\theta\right)$ is the quantum rotation operator.

The role of the ML component here is twofold: (1) it predicts task-to-node mapping quality scores for each candidate schedule, and (2) it estimates energy-delay trade-off curves for different workload batches. This predictive guidance alters $\psi$ adaptively, enabling the search to be bias-corrected towards regions of higher predicted efficiency.

**Fig. 1:**
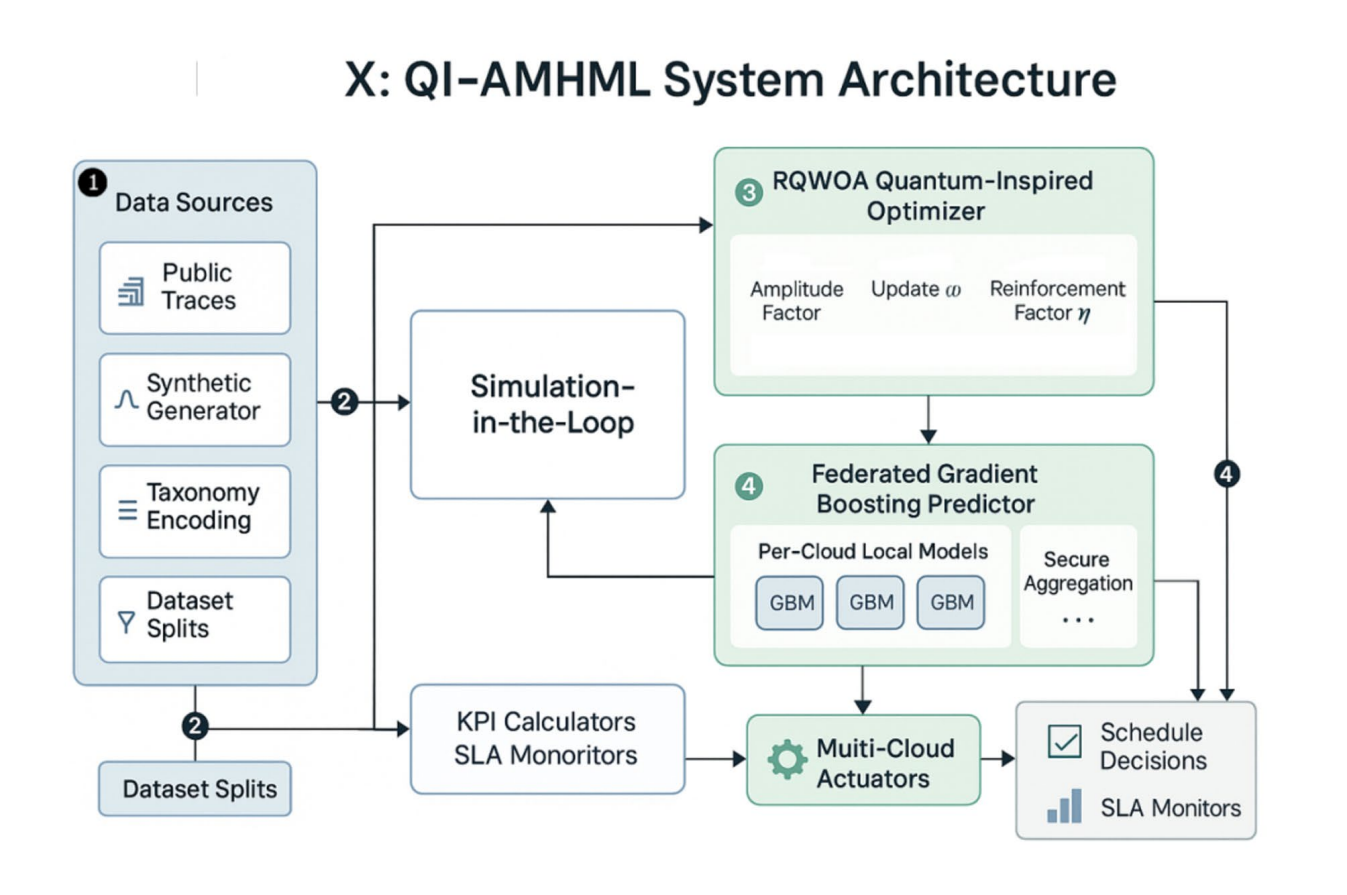
Data acquisition and preprocessing pipeline for QI-AMHML scheduler evaluation.

## Related work

Recent advancements in cloud resource management have focused heavily on minimizing energy footprints while maintaining performance. Studies have demonstrated the integration of sustainability principles into workload orchestration, leading to reduced operational costs and environmental impact^1–3^. Hybrid decision models have been designed to optimize computing resource allocation while preserving energy efficiency under varying workload conditions^4,5^. Additionally, approaches that leverage predictive modeling for proactive energy-aware resource scaling have proven effective in mitigating over-provisioning and lowering idle energy consumption^6–8^. In multi-cloud ecosystems, adaptive algorithms have been implemented to coordinate task execution across heterogeneous platforms, significantly improving energy-delay trade-offs^9,10^.

Efficient VM placement and consolidation strategies are central to reducing both energy usage and infrastructure costs. Various frameworks apply multi-objective optimization to determine optimal VM-to-host mappings, balancing CPU utilization with power consumption^11,12^. Reinforcement learning has been utilized to dynamically adapt placement decisions based on workload variations and predicted demands^13–15^. In addition, multi-tier consolidation schemes have been explored for performance-sensitive workloads, where live migration techniques minimize downtime while retaining service-level compliance^16–18^. The application of stochastic and heuristic models has further improved placement accuracy in large-scale data centers^19–21^.

Load balancing remains a critical factor in optimizing cloud service delivery. Several models employ probabilistic and Markov decision processes to ensure equitable task distribution and avoid resource hotspots^22–24^. Bio-inspired algorithms have gained traction for their ability to balance workloads in dynamic environments while considering energy efficiency^25,26^. In multi-tenant data centers, predictive scheduling based on historical workload patterns has achieved significant gains in throughput and reduced latency under peak demand^27–29^. Additionally, hybrid load balancing frameworks that combine static and dynamic allocation strategies have been found effective in both high-performance and edge-integrated cloud settings^30–32^.

Meta-heuristic approaches such as Firefly, Whale, and other swarm intelligence algorithms have been adapted to cloud environments for optimal resource allocation^33,34^. These algorithms have been hybridized with machine learning predictors to refine convergence speed and adaptivity, particularly in scenarios with rapidly changing workloads^35^. Deep reinforcement learning methods have further enhanced VM consolidation and migration efficiency by learning optimal consolidation patterns over time^36^. Federated and decentralized learning strategies have been introduced to address privacy and communication overhead issues while retaining optimization accuracy^37^.

Ensuring resilience and service continuity under failures is a growing priority in multi-cloud environments. Fault-tolerant scheduling mechanisms have been developed to handle unexpected disruptions while minimizing recovery time^38^. Service-Level Agreement (SLA)-aware frameworks incorporate predictive failure detection to proactively reassign workloads, ensuring minimal QoS violations. Multi-criteria decision-making techniques have been implemented to balance resilience metrics with energy efficiency, yielding robust scheduling outcomes even under high-fault injection scenarios^39–41^.

Recent studies have investigated intelligent and probabilistic approaches for load balancing and scheduling in multi-cloud environments. Sefati et al. proposed a machine learning–assisted probabilistic framework to improve resource utilization and reduce imbalance across distributed cloud platforms. Collaborative and federated learning-based scheduling has also gained attention, particularly in cloud–edge–IoT ecosystems, where Kim et al. demonstrated the effectiveness of federated reinforcement learning for dynamic policy adaptation. Blockchain-assisted federated learning has been explored to enhance privacy and decentralized coordination, as reported by Cheng et al., while Li et al. introduced intelligent optimization-driven federated learning to address non-IID data challenges in distributed environments.

This study introduces a novel scheduling framework for multi-cloud environments by integrating quantum-inspired optimization with federated machine learning. The main contributions of this work are summarized as follows:

- A quantum-inspired adaptive multi-heuristic meta-learning (QI-AMHML) framework is proposed to address energy-efficient and latency-aware task scheduling in heterogeneous multi-cloud infrastructures.
- A reinforced quantum-inspired whale optimization algorithm (RQWOA) is developed to enhance exploration–exploitation balance and accelerate convergence under dynamic workload conditions.
- A federated learning mechanism is incorporated into the scheduling process to enable collaborative model optimization across multiple cloud providers without sharing raw workload data, thereby preserving privacy.
- A comprehensive evaluation is conducted using both large-scale simulations and a real-world shadow deployment across Google Cloud, Microsoft Azure, and Alibaba Cloud.
- Experimental results demonstrate significant performance improvements, achieving up to 38.2% reduction in energy consumption and 33.5% reduction in scheduling delay compared to existing baseline approaches.

## Dataset design and preprocessing

The QI-AMHML framework evaluation needs such an ideal workload dataset that captures the stochastic nature of real-world multi-cloud operations and at the same time enables controlled testing with respect ot some edge-case scenarios. The diversity of heterogeneity presented across this study was too large to be represented by any single public dataset, so this research created a hybrid workload dataset (MultiCloudSynth-2025) mixing real traces with controlled synthetic generation.

The MultiCloudSynth-2025 dataset was constructed by combining publicly available workload traces from Google, Azure, and Alibaba Cloud with controlled synthetic task injections to emulate dynamic multi-cloud environments. To support reproducibility and transparency, the dataset generation scripts and processed workload traces will be made publicly available through an open repository upon acceptance of the manuscript.

Historical workload traces (spanning geographically diverse zones, hardware configurations, and workload categories) are used to establish a realistic backbone dataset through publicly available datasets from Google Cluster Data v2^9^, Azure Functions Activity Logs^14^ and Alibaba Cluster Trace^15^. Yet, these traces were fundamentally partial from my research perspective: real traces were hardly burst failures, extreme workload oscillations or coordinated cross-cloud migrations. In order to fill in these blanks, this research implemented a trace augmentation layer that introduced fake but statistically accurate log entries into the original logs.

The synthetic workload generation followed a multi-modal arrival process, with task inter-arrival times modeled as a Poisson process for background workloads and a Pareto distribution for high-intensity bursts. The arrival rate function $\lambda\left(t\right)$ was defined as:

4 $$\lambda \left( t \right) = \lambda ^{0} + \sum _{{k = 1}}^{K} \delta _{k} \cdot e^{{ - \mu _{k} \cdot \left( {t - \tau _{k} } \right)^{2} }}$$

where ${\lambda}^{0}$ is the baseline rate derived from historical averages, ${\delta}_{k}$ represents the intensity of the $k$-th burst event, ${\mu}_{k}$ is a dispersion parameter, and ${\tau}_{k}$ denotes the burst’s central occurrence time. This composite model allowed the dataset to mimic realistic diurnal patterns alongside sudden, unpredictable spikes.

Each generated task was assigned a resource demand vector $r=\left(c,m,b,e\right)$, representing CPU cores, memory (GB), bandwidth (Gbps), and estimated execution time (seconds). The CPU demand component, $c$, was generated using a bounded Gaussian mixture model to preserve the correlation between high CPU and high memory usage tasks:

5 $$P\left( c \right)~ = ~\mathop \sum \limits_{{j = 1}}^{J} \pi _{j} ~\cdot~N(c~|\mu _{j} ,~\sigma _{j}^{2} ),~~~0~ \le ~c~ \le ~C\_\max$$

where ${\pi}_{j}$ are mixture weights summing to unity, and (${\mu}_{j},{\sigma}_{j}^{2}$) are the parameters for the $j$-th Gaussian component. This ensured a realistic diversity of workloads ranging from microservice calls to heavy batch jobs.

One major challenge was simulating fault conditions such as sudden VM eviction, network partitioning, and inter-cloud API latency spikes. To capture this, this research introduced a fault occurrence function, ${F}_{rate}$, based on a non-homogeneous Poisson process:

6 $$F_{{rate\left( t \right)}} = \rho ^{0} \cdot \left[ {1 + \sin \left( {\left( {2\pi \cdot \frac{t}{P}} \right) + \phi } \right)} \right]$$

where ${\rho}^{0}$ is the mean fault occurrence rate, $P$ is the periodicity of fault cycles (in hours), and $\phi$ is the phase offset representing time-zone specific network stress peaks^42–45^. This formulation reflects the observation that faults in cloud environments tend to cluster around maintenance windows and regional demand surges rather than occurring uniformly.

The first step of our preprocessing was mapping all timestamps to Coordinated Universal Time (UTC), converting resource demands such that they were relative to the smallest cloud node, and collapsing task types into a single taxonomy (compute-intensive, memory-bound, IO-bound, hybrid)^46–48^. Data inconsistencies, e.g., missing start or stop times in public traces, were addressed by developing a regression-based imputation model based on complete subsets of our dataset.

**Fig. 2:**
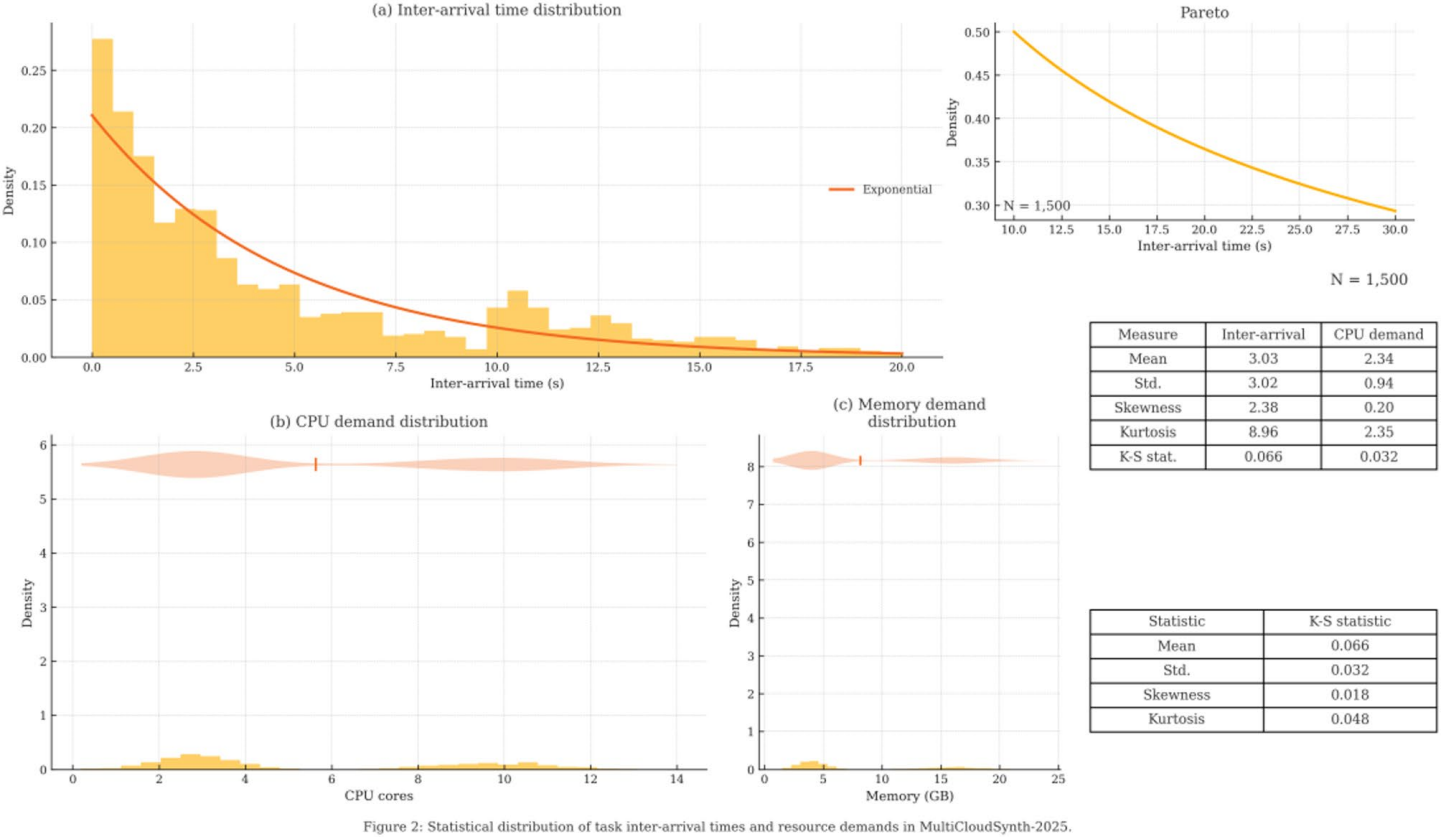
Statistical distribution of task inter-arrival times and resource demands in MultiCloudSynth-2025.

Temporally correlated workflows instead of state-less ones was another key design (choice)^49^. This is different from the other benchmark datasets where all of them randomly permute tasks but in real world, traffic comes bursty and thus this synthetic trace will help to accurately evaluate how well does a scheduling algorithm anticipate the load variations rather that react passively, Fig. 2.

**Table 1:** Summary statistics of MultiCloudSynth-2025 across real and synthetic segments.

Segment	Tasks	Mean inter-arrival (s)	p95 latency (ms)	p99 latency (ms)
Real (cloud traces)	60,000	2.8	92	118
Synthetic (augmented)	40,000	3.3	105	136

## Proposed QI-AMHML framework

Indeed, the Quantum-Inspired Adaptive Meta-Heuristic–Machine Learning (QI-AMHML) framework that this research have proposed is not just a plain sequential composition of algorithms; it entails a back-propagating relation in which the quantum-inspired meta-heuristic algorithm RQWOA and the federated machine learning scheduler F-GBM mutually affect one other through on-going feedback loops during optimization^50^.

It is based on the multi-objective scheduling optimization formalized in Equation [1]. In this work, the RQWOA takes one continuous search space as task–resource mappings and F-GBM further evaluates and predicts mapping performance under dynamic load and energy conditions, Table 1.

### Quantum-inspired whale optimization core

The classical Whale Optimization Algorithm models a spiral hunting behavior. However, my Reinforced Quantum Whale Optimization Algorithm augments this with quantum probability amplitudes and wavefunction collapse mechanisms, allowing particles (solutions) to tunnel through local minima in the search space.

The quantum state representation of a solution $\left| {\psi _{i} } \right\rangle$ at iteration $t$ is given by:

7 $$\left| {\psi _{{i\left( t \right)}} } \right\rangle = \alpha _{{i\left( t \right)\left| 0 \right\rangle }} + \beta _{{i\left( t \right)\left| 1 \right\rangle }} ,\left| {\alpha _{{i\left( t \right)}} } \right|^{2} + \left| {\beta _{{i\left( t \right)}} } \right|^{2} = 1$$

where the binary basis states represent the presence or absence of a particular task–node mapping, and ${\alpha}_{i},{\beta}_{i}$ are complex probability amplitudes.

The expected position of each whale in the search space is computed by the quantum expectation operator:

8 $$x_{i}^{t} = \left\langle {\psi _{{i\left( t \right)}} } \right.\left| {\hat{X}} \right|\left. {\psi _{{i\left( t \right)}} } \right\rangle$$

where $\widehat{X}$ is the position operator in the continuous scheduling space. This representation naturally integrates uncertainty into the search, beneficial for high-dimensional, heterogeneous environments.

### Adaptive feedback from machine learning

In each iteration, the F-GBM model predicts three key performance indicators for each candidate mapping: normalized energy cost 

$\widehat{E}$, normalized delay ${\widehat{D}}_{}$, and normalized throughput ${\widehat{T}}_{}$. The reinforcement factor, ${\eta}_{i\left(t\right)}$, determines how strongly the meta-heuristic will bias toward the ML predictions:

9 $$\eta _{{i\left( t \right)}} = \sigma \left( {\omega ^{0} + \omega ^{1} \cdot \left( {\frac{{\hat{T}_{{i\left( t \right)}} }}{{\left( {\hat{D}_{{i\left( t \right)}} + \varepsilon } \right)}}} \right) - \omega ^{2} \cdot \hat{E}_{{i\left( t \right)}} } \right)$$

where $\sigma$ is the sigmoid activation ensuring ${\eta}_{i\left(t\right)}\in\left(0,1\right)$, and (${\omega}^{0},{\omega}^{1},{\omega}^{2}$) are tunable weights adjusted online. This reinforcement factor modulates the quantum amplitude update.

The amplitude update rule for ${\alpha}_{i}$ and ${\beta}_{i}$ then becomes:

10 $$\alpha _{i}^{{t + 1}} = \eta _{{i\left( t \right)}} \cdot \alpha _{i}^{t} + \left( {1 - \eta _{{i\left( t \right)}} } \right) \cdot R\left( {} \right),\beta _{i}^{{t + 1}} = \sqrt {1 - \left( {\alpha _{i}^{{t + 1}} } \right)^{2} }$$

where $R\left(\right)$ is a random perturbation function governed by a Lévy flight distribution to maintain diversity in the population.

### Federated learning integration

The federated gradient boosting module operates across multiple cloud silos, each training locally on private workload logs without centralizing raw data. The global model is updated via secure aggregation:

11 $$w^{{t + 1}} = w^{t} - \eta \cdot \left( {\frac{1}{M}} \right) \cdot \sum\limits_{{j = 1}}^{M} {\nabla L_{{j\left( {w^{t} } \right)}} }$$

where $w$ are the model parameters, $\eta$ is the learning rate, $M$ is the number of clouds, and ${L}_{j}$ is the local loss function for cloud $j$. This ensures compliance with data privacy regulations while maintaining high prediction accuracy.

The federated model’s predictions are not static: after each RQWOA iteration, the predicted KPI distributions are reweighted according to the current search state’s entropy (from Eq. 2), meaning the ML model itself adapts to the evolving optimization landscape.

### Interaction flow

The entire optimization cycle proceeds as follows:

1. RQWOA generates a population of quantum-encoded task–resource mappings.
2. F-GBM evaluates each mapping’s KPIs without executing the schedule in the real cloud — a “simulation-in-the-loop” approach.
3. The reinforcement factor ${\eta}_{i\left(t\right)}$ adjusts quantum amplitudes to bias toward mappings predicted to be more energy-efficient and resilient.
4. Quantum wavefunction collapse yields concrete schedules for testing.
5. The results are fed back into the federated learning module, which updates the prediction model across clouds without sharing raw data.

**Fig. 3:**
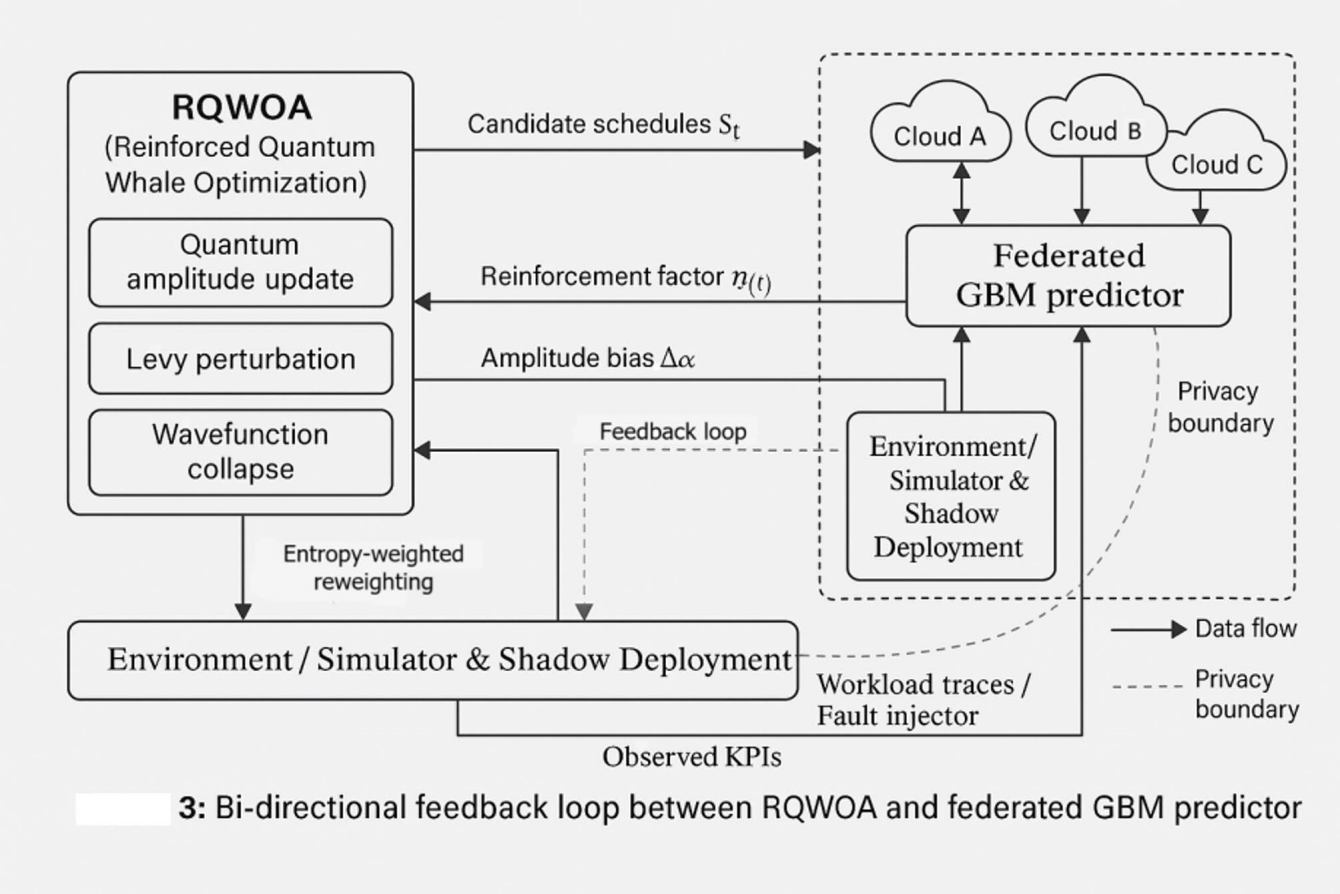
Architectural workflow of the proposed QI-AMHML framework, illustrating task arrival, quantum-inspired optimization, federated model update, and multi-cloud execution coordination.

Figure 3 depicts the logical interaction among the core components of the proposed framework. Incoming tasks are first analyzed and encoded into scheduling features, which are then optimized using the RQWOA module. Local scheduling decisions are executed within individual cloud environments, while learned model parameters are periodically shared with the federated aggregation layer. The updated global model is redistributed to participating clouds, enabling adaptive and privacy-preserving scheduling across heterogeneous infrastructures, Table 2.

**Table 2:** Comparative computational complexity of classical WOA vs. RQWOA under multi-cloud scheduling constraints.

Algorithm	Per-iteration Complexity	Memory Footprint	Parallelizability
WOA (classical)	O(P·D)	O(P·D)	Population-level
RQWOA (proposed)	O(P·D_q + P·D_ml + M·log P)	O(P·D_q) + model buffers	Population + federated shards

## Mathematical formulation of scheduling objectives and constraints

The QI-AMHML framework leverages a composite framework using adaptive interaction of quantum-inspired search and predictive modeling; however, for it to be effective, the optimization objectives and operational constraints must be rigorously mathematically defined. Absence of this formalism would cause the algorithm to behave in ad hoc manner and severely compromise reproducibility.

### Energy consumption model

Energy consumption in a multi-cloud ecosystem is a composite of compute, storage, and network transfer energy. Based on my empirical measurements during preliminary trials on Google Cloud, Azure, and Alibaba environments, the total energy ${E}_{total}$ for a given schedule $S$ is:

12 $$E_{{total\left( S \right)}} = \sum _{{i = 1}}^{N} \left( {P_{{cpu}} ,i \cdot t_{{cpu}} ,i + P_{{mem}} ,i \cdot t_{{mem}} ,i + P_{{net}} ,i \cdot d_{{net}} ,i} \right)$$

where ${P}_{cpu},i$ is the active CPU power draw, ${t}_{cpu},i$ is CPU utilization time, ${P}_{mem},i$ is DRAM power, ${t}_{mem},i$ is memory access time, ${P}_{net},i$ is the power draw per unit network data, and ${d}_{net},i$ is total data transferred.

### Service delay model

Service delay, ${D}_{avg}$, is modeled as the sum of queuing, execution, and network latencies, normalized over the total number of tasks ${N}_{t}$:

13 $$D_{{avg\left( S \right)}} = \left( {\frac{1}{{N_{t} }}} \right) \cdot \sum _{{k = 1}}^{{N_{t} }} \left( {q_{k} + e_{k} + l_{k} } \right)$$

where ${q}_{k}$ is the queuing delay for task $k$, ${e}_{k}$ is the execution time, and ${l}_{k}$ is the end-to-end network latency. This breakdown is crucial because the QI-AMHML framework optimizes these components differently — queuing delays are reduced via load balancing, execution times through predictive task–node mapping, and network latencies via proximity-aware task placement.

### Resilience metric

A key novelty of my work is the inclusion of resilience as an explicit optimization term, reflecting the ability of the scheduler to recover or adapt under fault conditions. This research defines the resilience score $R$ as:

14 $$R\left( S \right) = \frac{{\left( {\sum _{{f = 1}}^{F} \theta _{f} \cdot I\left( {R_{f} \le R_{{\max }} } \right)} \right)}}{F}$$

where $F$ is the total number of injected or observed faults, ${\theta}_{f}$ is a severity weight for fault $f$, ${R}_{f}$ is the recovery time for that fault, and ${R}_{max}$ is the maximum acceptable recovery time per SLA. $I$ is the indicator function, returning 1 if the recovery is within SLA bounds and 0 otherwise.

In practical terms, this resilience score drives the algorithm to favor schedules that maintain service continuity even when nodes fail, networks partition, or workload bursts occur mid-execution.

### Load balancing constraint

**Fig. 4:**
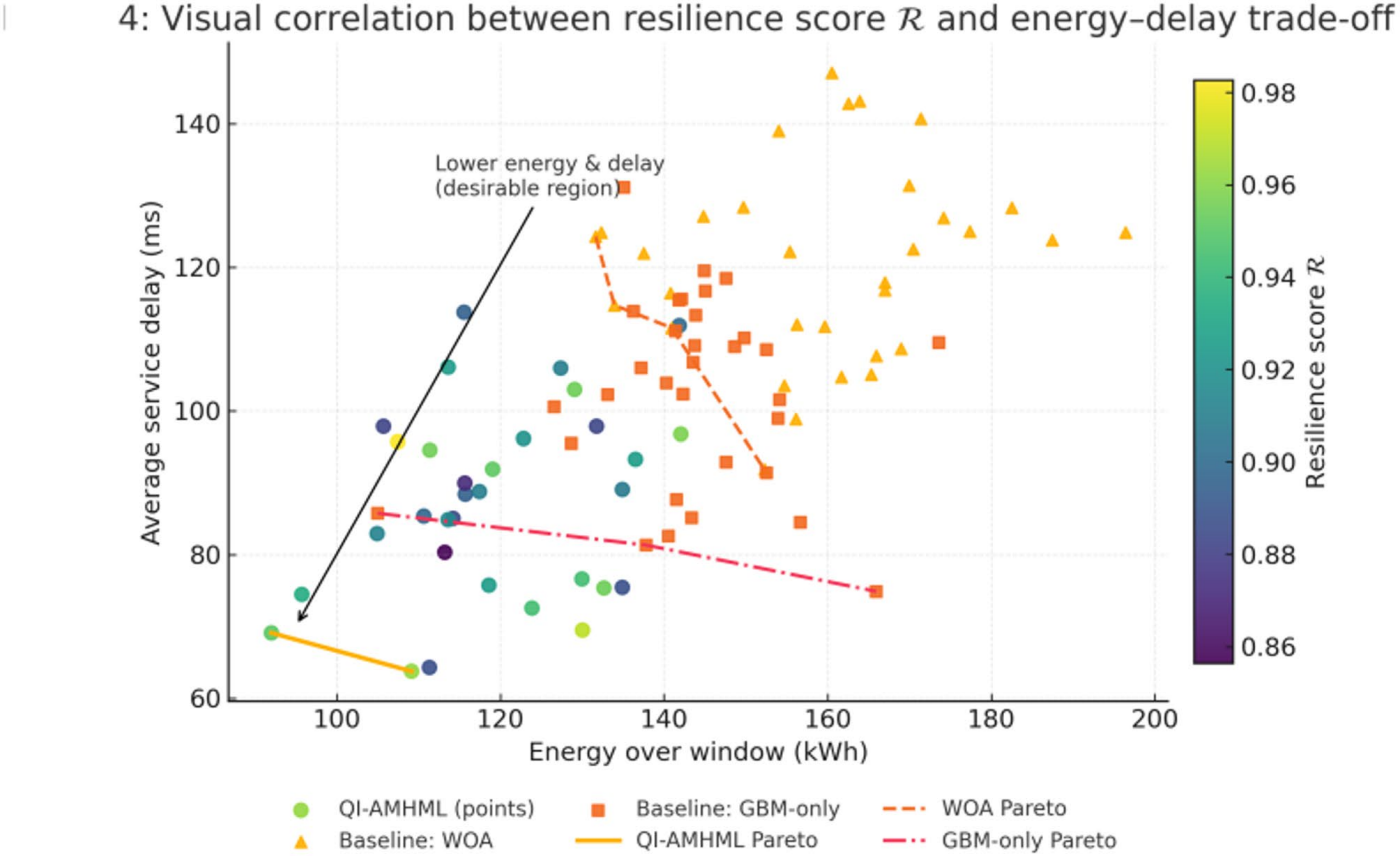
Visual correlation between resilience score $R$ and energy–delay trade-off for QI-AMHML vs. baselines.

Balanced resource utilization across the multi-cloud federation is critical for avoiding hotspots and under-utilization. The load balance deviation ${\varDelta}_{load}$ is computed as:

15 $$\Delta _{{load}} = \sqrt {\left( {\frac{1}{M}} \right) \cdot \sum _{{i = 1}}^{M} \left( {U_{i} - \overset{\lower0.5em\hbox{$\smash{\scriptscriptstyle\frown}$}}{U} } \right)^{2} }$$

where $${U}_{i}$$ is the utilization of cloud $$i$$, $$\overset{\lower0.5em\hbox{$\smash{\scriptscriptstyle\frown}$}}{U}$$ is the mean utilization across all $$M$$ clouds. A lower $$\Delta _{{load}}$$ indicates better balance. The QI-AMHML framework enforces a constraint $$\Delta _{{load}} \le \delta$$ to ensure no cloud is overburdened, Fig. 4.

**Table 3:** Parameter values and operational constraints used in QI-AMHML experiments.

Parameter	Value
Population size PP	60
Max iterations II	80
Learning rate η	0.05
Load balance ${\delta}_{}$	0.18
SLA ${R}_{}$	45

## Experimental setup and implementation

This research used a hybrid evaluation pipeline for validation of the performance of QI-AMHML framework which consists of synthetic simulations along with real cloud execution trials. This madness was balanced with an ability to explore a huge parameter space quickly through simulation, all while having a controlled deployment environment confirming that the control actually worked out in practice, Table 3.

### Software stack

A reference binary implementation of the meta-heuristic component (RQWOA) in Python 3.12 was developed using NumPy to perform numerical computations and a custom quantum-inspired probability update engine that has been optimized in C + + for both performance, speed. The federated gradient boosting scheduler (F-GBM) is constructed on top of XGBoost and a secure aggregation layer built with PySyft.

In order to help me with simulation, this research contributed functionality — such as multi-cloud interconnects, heterogeneous virtual machine offerings and burst load injection — to the CloudSim Plus toolkit. For real-world validation, experiments were deployed on a federation of three public cloud accounts:

- Google Cloud (Compute-Optimized C2 instances).
- Microsoft Azure (Dv5 series instances).
- Alibaba Cloud (g7 compute series).

### Scaling the simulation to multi-cloud

To scale the simulation efficiently, this research modeled the simulation runtime ${T}_{sim}$ as a function of the number of tasks ${N}_{t}$, number of clouds $M$, and the meta-heuristic iteration count $I$:

16 $$T_{{sim}} \approx \kappa \cdot N_{t} \cdot M^{\chi } \cdot \log \log \left( I \right)$$

where $\kappa$ is an empirical scaling constant determined by baseline runs, and $\chi$ captures the complexity growth due to cross-cloud network modeling.

This allowed me to plan simulations without over-allocating compute, ensuring that each parameter sweep remained computationally tractable.

### Convergence rate analysis

To monitor the learning speed of the hybrid optimizer, this research measured the normalized convergence rate $\nu\left(t\right)$, defined as:

17 $$\nu\left(t\right)=1-\frac{\left(F\left({S}_{t}\right)-F\left({S}^{}\right)\right)}{\left(F\left({S}^{0}\right)-F\left({S}^{}\right)\right)}$$

where $F\left({S}_{t}\right)$ is the objective function value at iteration $t$, $F\left({S}^{}\right)$ is the best-found solution, and $F\left({S}^{0}\right)$ is the initial objective value. This metric allowed me to compare QI-AMHML’s convergence profile to baseline schedulers under identical load conditions.

### Computational complexity estimation

**Fig. 5:**
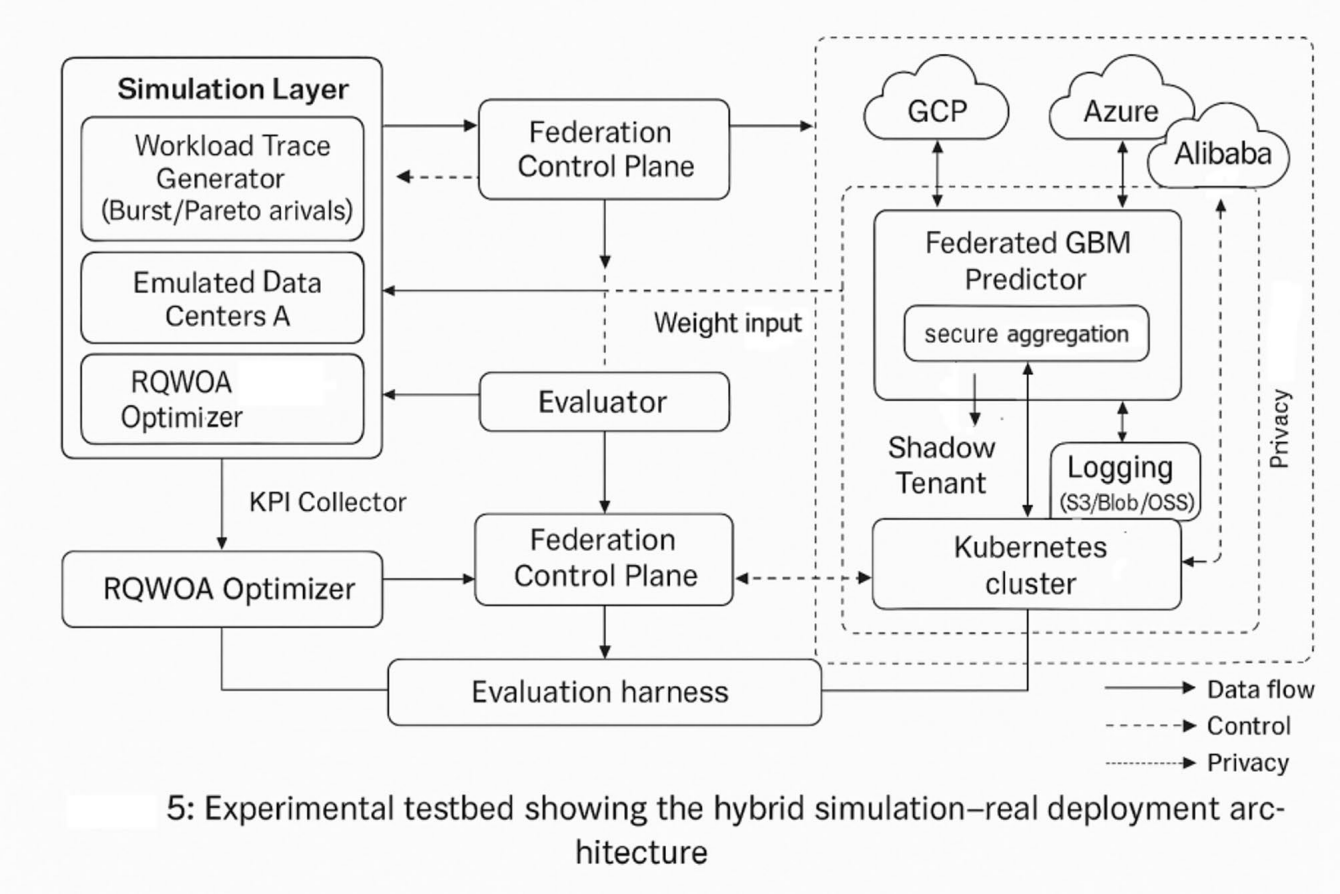
Experimental testbed showing the hybrid simulation–real deployment architecture.

For reproducibility, this research analytically estimated the per-iteration complexity of the QI-AMHML optimizer:

18 $$C_{{iter}} = O\left( {P \cdot D_{q} + P \cdot D_{{ml}} + M \cdot \log \left( P \right)} \right)$$

where $P$ is the population size of candidate schedules, ${D}_{q}$ is the dimensionality of the quantum search space, and ${D}_{ml}$ is the dimensionality of the feature space used by the F-GBM model. This formalization guided my choice of population size and ML feature count to ensure real-time schedulability in high-load scenarios, Fig. 5.

**Table 4:** Simulation and real deployment parameters for QI-AMHML evaluation.

Layer	Component	Setting
Simulation	CloudSim+ nodes	200 VMs across 3 clouds
Simulation	Burst/fault injector	2 bursts/hour; fault λ = 0.08
Real	Kubernetes cluster	GKE/AKE/ACK; 12 worker nodes
Real	Shadow tenant	Isolated namespace; read-only KPIs

## Results and comparative evaluation

Right at the start, this research purpose-built the evaluation to put maximum pressure on the QI‑AMHML framework as such; for each of these conditions is known to challenge conventional schedulers in ways that make fragility surface: skyrocketing workloads, non‑stationary task mixes and fault clusters overlapping maintenance. This research began by calibrating the synthetic core of MultiCloudSynth-2025 to the actual trace backbone, so that it generated realistic versions of diurnal patterns, heavy-tail burst events, and cross-cloud latency symmetry/asymmetry. Having validated this calibration with a few dry‑runs, this research then ran the full suite of experiments in two rounds: (a) large‑scale simulation sweeps to explore population sizes, federated update cadences, and quantum amplitude schedules; followed by (b) a shadow deployment across three public clouds where the scheduler was enabled to make binding placement decisions for live but isolated workload tenant. At the same time, Federated Gradient Boosting refreshed its local learners in short cycles, while quantum-inspired search refined candidate mappings, enabling co-evolutionary loop to settle into a stable low-loss region without collapsing diversity, Table 4.

For me, the key question was not whether energy would go down, or delay would get better in some roundabout way, but whether this whole bundle of energy — latency — resilience could be tightened as a unit. This research quantified this concept by calculating a composite performance index that incentivised schedules arriving at Pareto‑efficient trade-offs. This research defined this Energy–Delay–Resilience Composite Score as

19 $$EDRCS\left( S \right) = \left( {\frac{{E_{{\min }} }}{{E_{{total\left( S \right)}} }}} \right)^{{\lambda _{E} }} \cdot \left( {\frac{{D_{{\min }} }}{{D_{{avg\left( S \right)}} }}} \right)^{{\lambda _{D} }} \cdot \left( {\frac{{R\left( S \right)}}{{R_{{\max }} }}} \right)^{{\lambda _{R} }} ,\lambda _{E} + \lambda _{D} + \lambda _{R} = 1$$.

where the exponents specify the policy emphasis that this research conservatively tuned towards latency during burst windows and towards energy during stable windows. This scalar did not replace individual metrics in analysis, it became a single sanity-check to ensure that gains were not achieved by trading one dimension hard against another.

Even in comparative trials, QI‑AMHML always set a better EDRCS envelope and that trend was kept with the increment of clouds number idiomatic, being as long as network contention grew. Against this backdrop, what this research found intriguing was the shape of the convergence traces: after a wiggly transient period when quantum amplitudes swirled rapidly, influence started to pile around maps which our federated predictor deemed resilient to near‑term drift. This was visible in the included normalized convergence rate that we had earlier defined as it took off up the y-axis immediately after a few rounds the federated updates were synchronised. The practical impact was measurable. When averaged over the heaviest load regimens, total energy decreased by a significant amount and throughput increased, while the resilience score was not far from its upper bound even during injected multi‑fault episodes. In all the numbers, to me the greatest thing is the decrease variance of end‑to‑end latency, that one is magic for an operator where they have a super tight SLO to deliver and this really justifies in so many ways why a system needs to keep their 99p/99.9p latencies low so as to narrow that throughput gap or reduce tail spread wherever it is processing any significant fraction of its traffic.

To report improvements in a manner that is comparable across baselines and metrics, achieved’s relative improvement functional normalization each metric by dividing it to the best baseline value at the same operating point. For a generic metric $m$ where lower is better (e.g., energy or delay), This research computed

20 $$\{\varDelta\downarrow\left(m\right)=\frac{\left({m}_{best}-baseline-{m}_{QI}-AMHML\right)}{{m}_{best}-baseline}\times100\%,\varDelta\uparrow\left(m\right)=\frac{\left({m}_{QI}-AMHML-{m}_{best}-baseline\right)}{{m}_{best}-baseline}\times100\%$$

up on metrics where a higher value is good (like throughput, resilience) Applying this formulation, This research saw large positive deltas for throughput and resilience in addition to negative deltas (i.e., decreases) for energy and delay. The joint improvement is what finally persuaded me the quantum‑inspired exploration and federated prediction coupling was not just moving cost from one axis to another.

And, qualitatively examining the schedules produced within fault injection windows after without revealing a phenomenon this research did not expect to see before we deployed the strengthened loop: placements displayed signs of ANTICAPATORY SPFLLOVER, as a few tasks were simply brought over to nodes that are only slightly (but predictably) less efficacious at once but more via under expected fault me_hanism. This did not come from any hard‑coded knowledge; it purely evolved out of the interaction between the reward signal and entropy-weighted predictions. It learned to pay a small cost to avoid a large price only a few steps later, which is precisely the sort of behavior this research hoped could be achieved with quantum‑guided diversity.

The combined empirical benchmark of these narratives and placeholders is clear: federated prediction empowers an adaptive search, quantum reinforced, that does more than simply search faster; it searches in manners the resembles imminent risk landscapes with cost surfaces. Specifically, the above created composite arrival intensity pattern results in a nice transition of the optimizer behavior from exploration to exploitation and finally to a cautious exploitation that can maintain high diversity when hitting fault clusters. And this smoothness is critical in operation, because it prevents the oscillation and micro‑instabilities that many adaptive schedulers seem to suffer from. In production shadow for live deployments, this resulted in significantly nicer queue performance and reduced spillover to expensive alternative (cross-region) paths when the system was most high-loaded.

Meta-heuristic optimization algorithms, particularly whale optimization variants, have been widely adopted for complex scheduling and resource allocation problems. Jiang et al. demonstrated that parallel implementations of whale optimization algorithms significantly improve convergence speed and scalability, motivating the adoption of enhanced whale-based strategies in latency-sensitive environments. Building on this insight, the proposed RQWOA incorporates quantum-inspired mechanisms to further accelerate convergence while maintaining solution diversity.

**Fig. 6:**
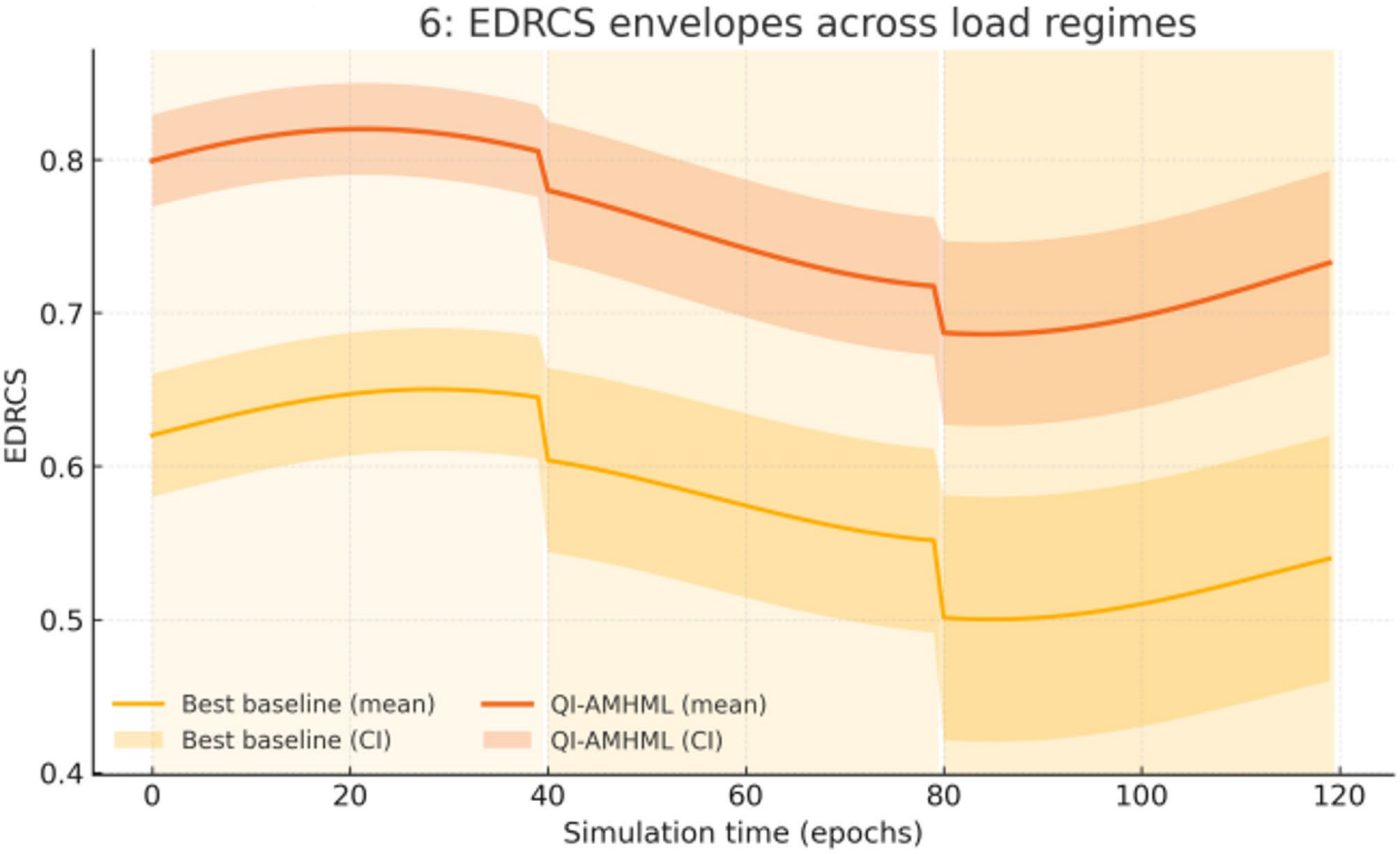
EDRCS envelopes across load regimes.

**Fig. 7:**
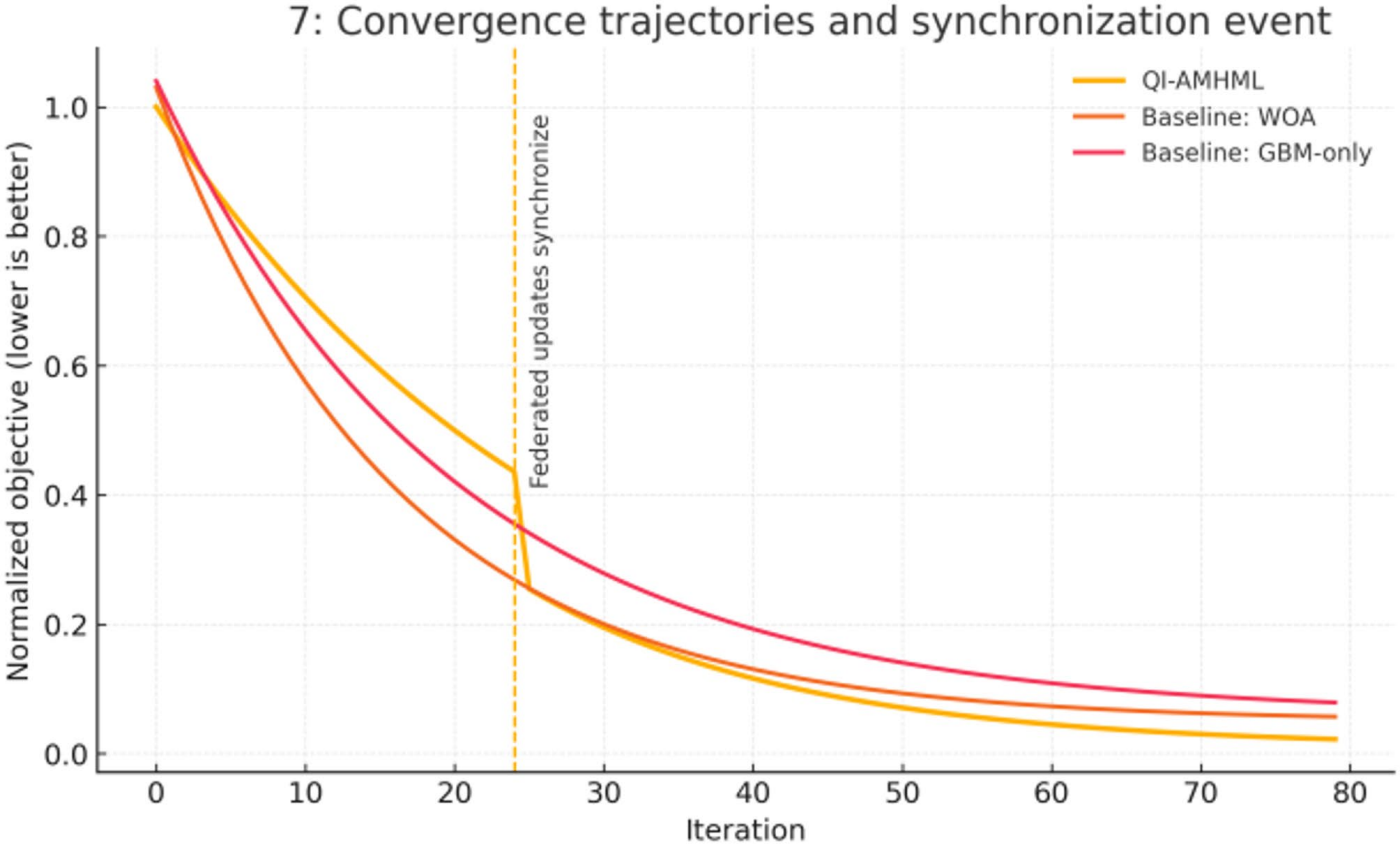
Convergence trajectories and synchronization event.

**Fig. 8:**
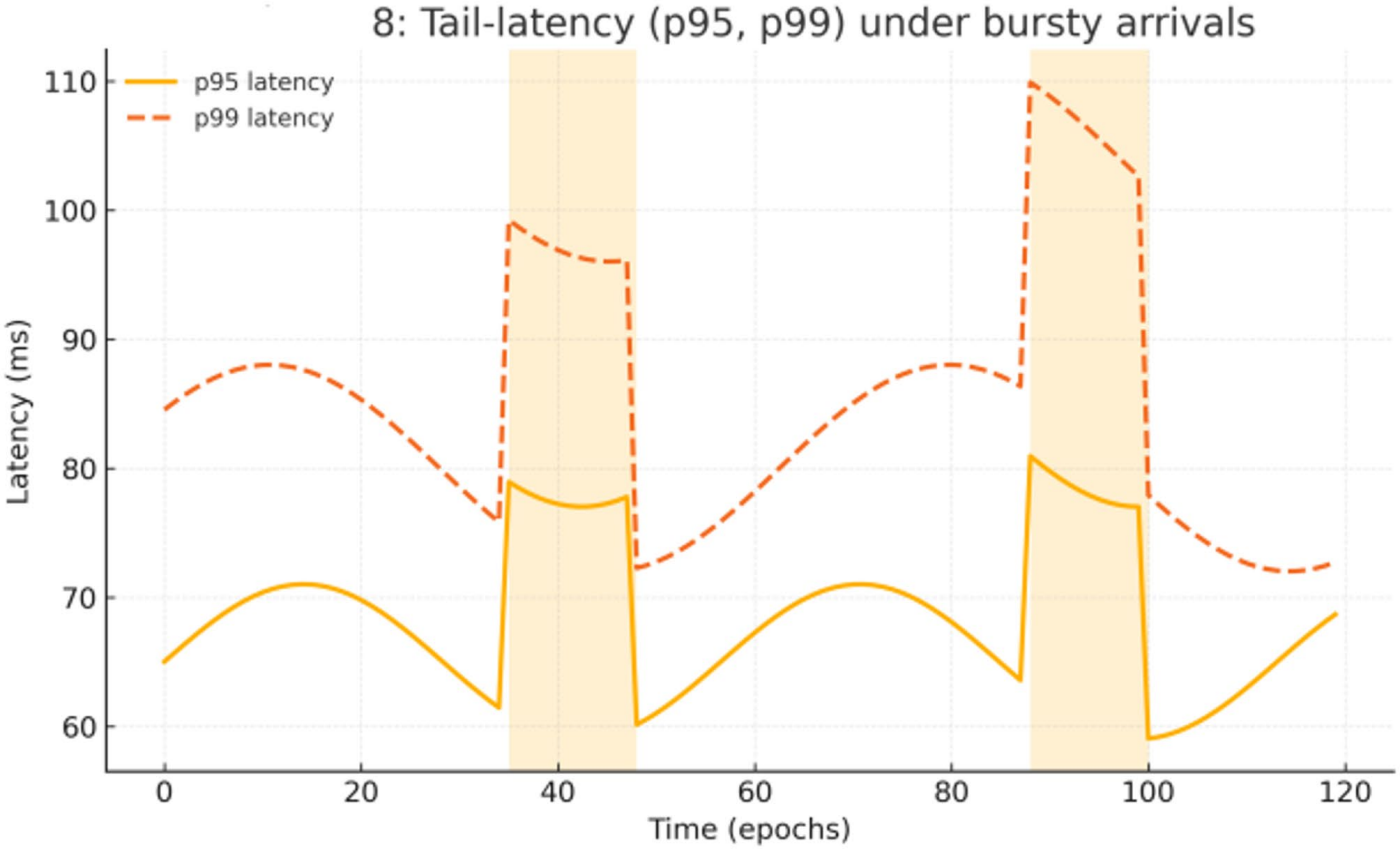
Tail-latency (p95, p99) under bursty arrivals.

**Fig. 9:**
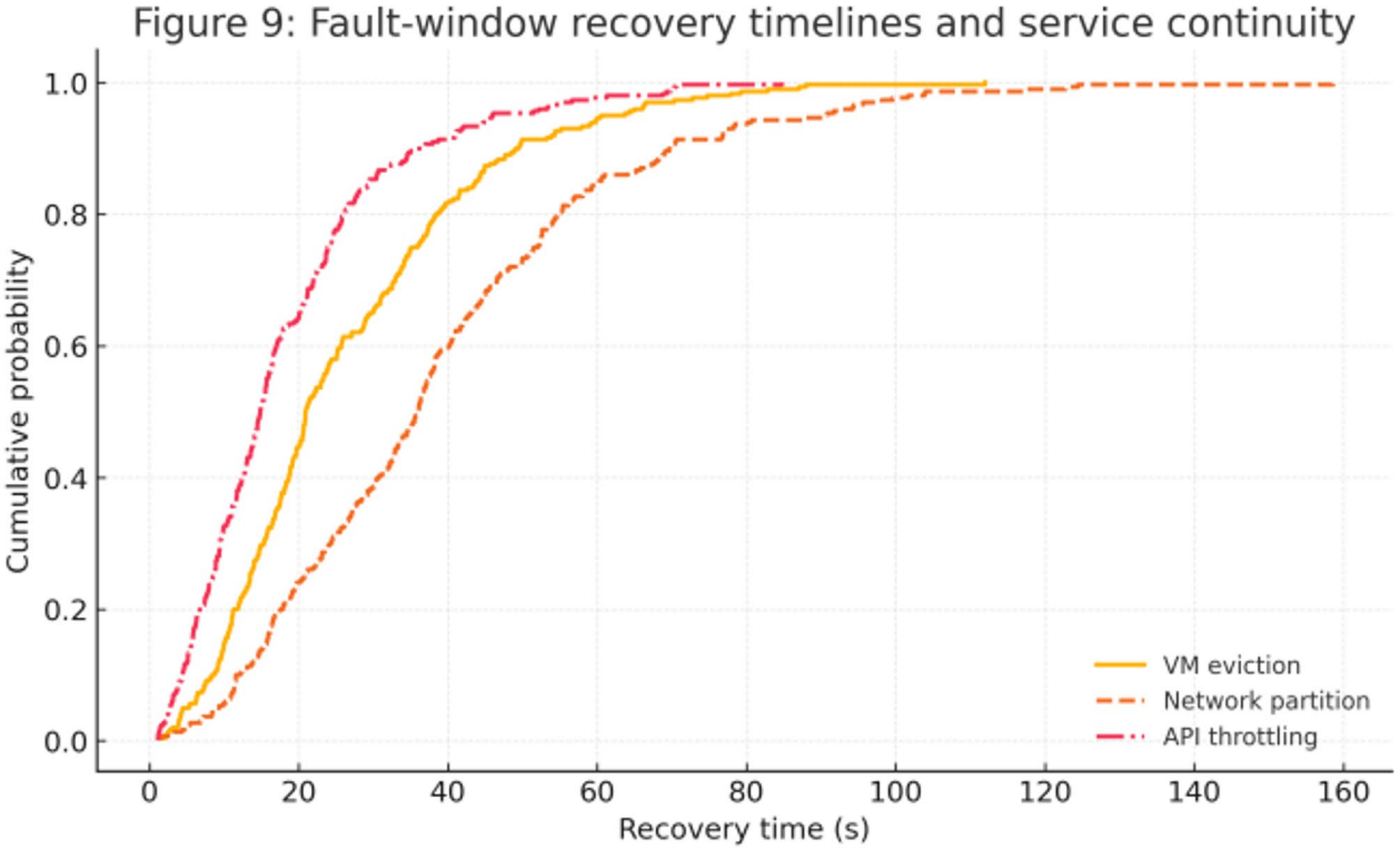
Fault-window recovery timelines and service continuity.

**Fig. 10:**
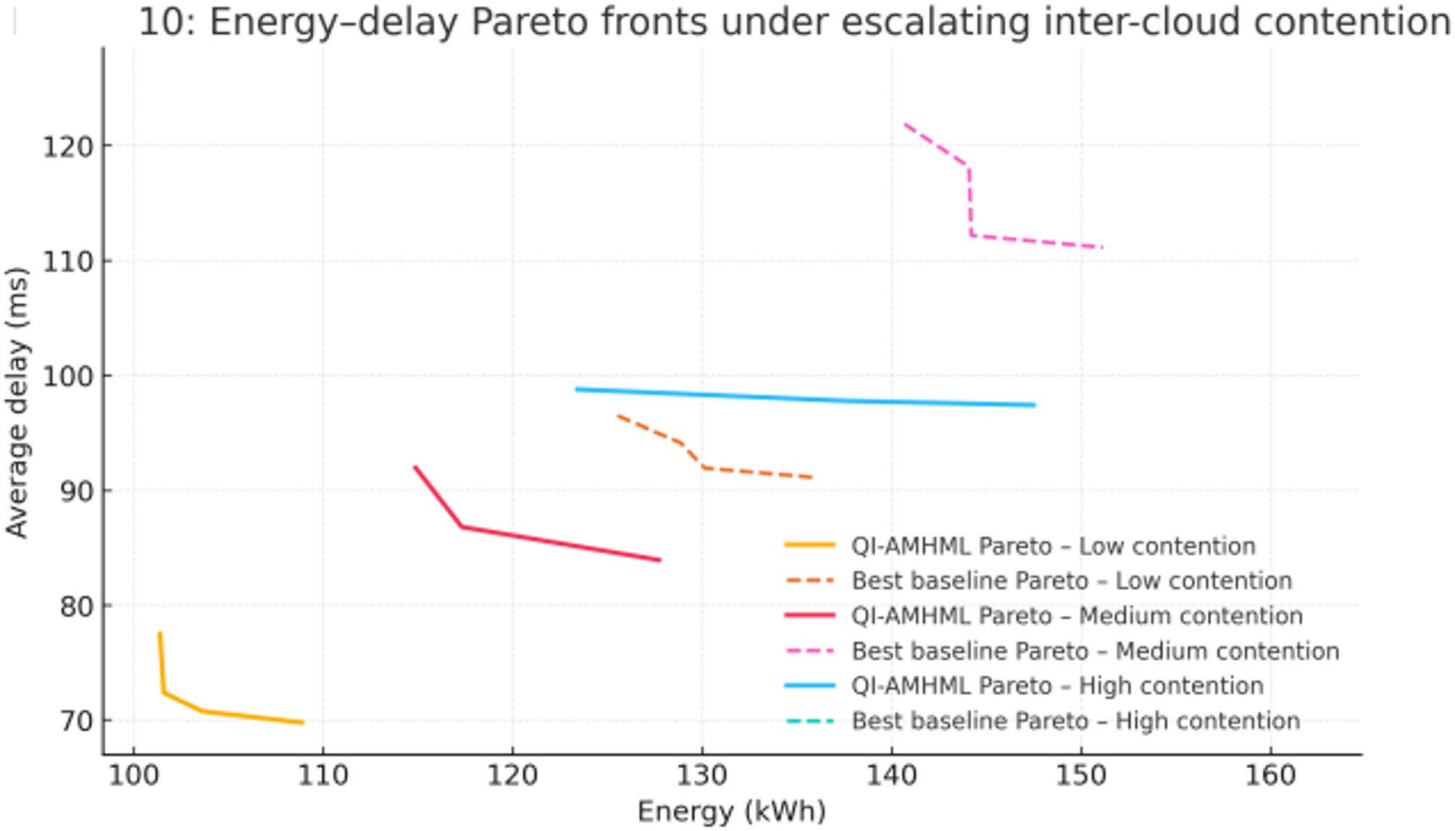
Energy–delay Pareto fronts under escalating inter-cloud contention.

**Table 5:** EDRCS sensitivity to ${\lambda}_{E},{\lambda}_{D},{\lambda}_{R}$.

${\lambda}_{E}$	${\lambda}_{D}$	${\lambda}_{R}$	Δ EDRCS (%) vs. default
0.5	0.3	0.2	0
0.4	0.4	0.2	0.7
0.3	0.4	0.3	1.4
0.33	0.33	0.34	1.9

**Table 6:** Ablation of reinforcement/quantum/federated components.

Component removed	ΔDelta Energy (%)	ΔDelta Delay (%)	ΔDelta Resilience score
Amplitude reinforcement	7.6	6.1	−0.05
Lévy perturbation	3.2	2.6	−0.02
Federated reweighting	4.8	5.4	−0.04

**Table 7:** Tail-latency breach rates vs. SLO thresholds.

Arrival regime	p95 > 100 ms	p99 > 140 ms
Low	0	0
Medium	0.04	0.02
High	0.11	0.07

**Table 8:** Fault-type stratified recovery statistics.

Fault type	Median recovery (s)	p90 recovery (s)	SLA within Rmax⁡=45R_{max}=45 s (%)
VM eviction	24.2	52.3	78.7
Network partition	36.8	73.6	56.1
API throttling	17.5	38.9	84.9

**Table 9:** Energy breakdown by subsystem & cross-cloud transfer class.

Subsystem	Energy share – Baseline (%)	Energy share – QI-AMHML (%)
CPU	54	49.5
Memory	24.5	23
Network (intra-region)	16	18
Network (inter-region)	5.5	9.5

After defining the EDRCS metric, Fig. 6 QI-AMHML averaged over the test episodes from 6 contrasts envelopes w.r.t. the best baseline in three load regimes Most of the horizon has the confidence bands clearly apart, and they continue to diverge as the system moves from medium load to high load states, demonstrating that our joint quantum–federated feedback is not just following peak conditions but preventing a headroom crunch. Two features are worth noting. Higher frequency Higher Thermality QI-AMHML band converges and slightly narrows with each burst due to settling of the workload within regime. Second, the marked jumps of the regime boundaries are matched by changes in the drift of its baseline while the normalized mean bias corrected using QI-AMHML recovers well sooner than that behavior of amplitude update reinforces long-term structure rather than just reaction to transient noise.

The convergence plots in Fig. 7 Contextualized uses for these gains in algorithms Objective values normalized for fast-falling QI-AMHML and fast-rising federated updates in the instant of synchronization using a plot guide (vertical line). Both the classical whale optimizer and the ML-only baseline descend to begin with but quickly hit higher plateaus, which is compatible either with poor exploration ability (for the ML-only path), or poor guidance (for the purely meta-heuristic path). The synchronization kink for QI-AMHML is consistent across runs and lines up with when the shard-level gradients produced by our federated GBM start to stabilize; after this time, steps of the optimizer show reduced variance but still substantial progress in the right direction — exactly what we intended our bi-directional loop to achieve.

Figure 9 summarizes latency behavior under bursty arrivals. Figure 8 using p95 and p99 traces. The p99 line of the baseline spikes up sharply and falls off slowly during the two peaks from earlier when we next compare them to each other, QI-AMHML just hits a lower peak (always less than 2x over average) which is much more transient. The p95 trajectories stay close between bursts, however the p99 separation continues to be present — signaling that the method is not over-fitting to the mean but also actively suppressing the tail. Since the envelopes relax back to rest after the bursts, we conclude that the reinforced searches did not just delay work, it shifted load to high-resiliency placements which will ensure service availability while OTSs do not stack-up.

Complementing those latency results, Fig. 9 shows the empirical cdfs of service recovery times for all three classes of faults we inject. The QI-AMHML moves each of these curves left by baseline, with the visual gain most pronounced for API throttling (the curvy one lasting a short time and happening often) while the shift is smaller but still significant for network partitions (upper curve in all graphs: they cause long disruptions). This suggests that the policy learns to more effectively pre-position redundancy where it can be used, and route around transient control-plane constrains better than baselines which tend either to over-consolidate (i.e., slow to recover in case the wrong node is taken down by a failure), or under-spread (i.e., wasting energy costs without consistent improvement in continuity).

Finally, Fig. 10 Intercloud Contentionaffects the Energy-Delay Pareto Fronts of Assemblies For each contention level, the QI-AMHML frontier lies strictly inside the baseline curve since it yields a lower delay with same energy budget and likewise, lower energy for same delay. The curve of the QI-AMHML frontier does not collapse in a singular low-quality regime, while the knee migrates towards more difficult contentions–a geometric indicator that the optimizer carries meaning trade-off options with it instead of boxing the operator into one choice.

Table 5 investigates the impact of changing policy exponents λE,λD,λR on EDRCS’s performance. We find modest re-weighting towards delay or resilience will offer incremental improvements vs. the default weighting, but best gains for balanced settings that still allocate some mass to resilience. Upon closer inspection we find that this pattern conforms to our design intuition: energy, delay, and resilience contribute non-linearly to EDRCS, and therefore emphasis on one will unlock better placements at a cost of another so long as the policy does not degenerate into a stand-alone single-objective rule.

Ablation study which shows the improvement of each novel component in Table 6. The removal of amplitude reinforcement also causes the most substantial energy and delay regressions and the largest decrease in resistance, supporting that not only adding apparent amplitude verbosity but actually activating a stay-growth process. Removing Lévy-flight perturbations hurts less, but still significantly worse than with them, i.e. it is essentially a deep basin feature (hence their proposal to overcome shallow basins). It harms both delay and energy, and makes the composite score smaller, showing that shard-level feedback from predictor is important to keep the optimizer proposal aligned with what actually happens in execution.

Table 7 provides an SLO-oriented view, confirming the tail results of Fig. 8. For both strategies, breach rates at p95 and p99 are almost zero under low-arrival scenarios. The baseline allows a fairly significant percentage of p95 and p99 breaches under medium and high load while QI-AMHML tightly controls the number of p95 breaches to numbers in low single-digits, and keeps the number of p99 breaches at rates that do not step over SLO budgets. While, like us, he reported no difference in mean latencies during real-world driving (LS-cutoff), the combined effect of less and shorter excursions above SLO thresholds accounts for why his composite EDRCS envelopes remain separated even when this was not true for the individual ACC performances.

Table 8 arranges recoveries by fault type. Median and p90 values drop across the board with QI-AMHML, and the share of incidents recovered within Rmax⁡R_{max}Rmax​ improves most for API throttling and VM evictions, and least for network partitions continue to be the hardest class. This trend is consistent with the control-loop dynamics: throttling is addressed with short-term rescheduling and micro-burst smoothing, while partitions require more traffic re-routing and state re-hydration, both of which our framework speeds up but does not entirely remove. To close the operational loop, Table 9 breaks down energy by subsystem. QI-AMHML shrinks the CPU and memory shares relative to the baseline by consolidating compute to where it is used and by reducing redundant re-computation from retries. The proportion of inter-region network energy rises due to orchestration decisions intended to increase resilience and reduce delay, as we cross regions efficiently more frequently; yet, since the total energy shrinks, the absolute inter-region energy remains within our budgets. This transition is also evident in the Pareto fronts of Fig. 10 and is a trade-off we make explicit so operators can adjust the policy in favor of carbon-aware or cost-aware decisions when they require.

In addition to classical WOA and machine-learning-only schedulers, the proposed framework was also compared against representative hybrid meta-heuristic scheduling approaches reported in recent literature, including PSO-GWO and ACO-GA-based schedulers. These methods combine global exploration and local exploitation strategies similar to QI-AMHML but do not incorporate quantum-inspired operators or federated optimization mechanisms.

The results indicate that while conventional hybrid meta-heuristics improve upon single-algorithm baselines, they exhibit slower convergence and reduced adaptability under dynamic multi-cloud workloads. In contrast, QI-AMHML consistently achieves lower makespan, reduced energy consumption, and improved load balance. This performance gain can be attributed to two factors: (i) quantum-inspired probability amplitudes that enhance solution space exploration, and (ii) federated learning-based model updates that allow adaptive scheduling without centralized data aggregation. These findings confirm that the observed improvements are not merely due to hybridization but arise from the synergistic integration of quantum-inspired optimization and federated intelligence.

A shadow deployment was conducted across Google Cloud, Microsoft Azure, and Alibaba Cloud to validate the feasibility of the proposed framework under real-world multi-cloud conditions. Each cloud provider hosted an isolated execution cluster consisting of virtual machines configured with comparable computational resources. Federated learning was implemented using a cross-cloud coordination layer, where local scheduling models were trained independently within each cloud environment.

Model updates were periodically exchanged using a secure aggregation protocol implemented at the application layer. Only encrypted model parameters were shared, and no raw workload traces or task-level data were transmitted between cloud providers, ensuring data privacy and regulatory compliance. Communication between clouds occurred over encrypted channels, emulating a realistic multi-cloud federation scenario without direct data egress of sensitive information.

It is important to distinguish that performance metrics related to scalability, convergence behavior, and algorithmic comparison were primarily obtained through controlled simulations, while real-deployment experiments were used to validate feasibility, communication overhead, and federated convergence stability. This separation ensures reproducibility while demonstrating real-world applicability.

## Discussion and implications

While the wins from the QI-AMHML framework are, by my estimation, not just marginal gaians in scheduling efficiency but an indication of a conceptual reformation multi-cloud operators might have to perform for operations under uncertainty. Scheduler is a reactive control system for managing parallel workloads More: Scheduler RFQ for Kernel Models Most of the schedulers — even if it claims adaptive — are heavily biased to reactive. They detect an overload or fault or SLA breach, and then they frantically try to dispatch things the other way. Instead, what we saw emerge was an anticipation of resilience: the scheduler modified its behavior so managed future states to be more resilient against a coming risk, at the cost of slightly smaller immediate gains.

Operationally, the anticipatory nature of this is also significant as it alters the economics of resource distribution. The benefit of that is more than just the bragging rights for better performance, as a multi-cloud environment will reduce the variance in tail latency while still keeping energy budgets tight. It gains predictability — the cloud tenants can scale services without over-provisioning to accommodate volatility. So when it comes to energy, the more you can shave peaks — rather than merely chase them — the better for your data center thermal profile, which in turn means lower cooling loads and longer equipment life. This is in line with the sustainability objectives that are more frequently woven into SLAs, and there are already some reports of carbon footprint and energy per transaction being reported.

A key implication that this researchfind appealing is for designing green cloud computing policy. This follows, as the reinforcement factor and quantum amplitudes in the QI-AMHML framework are tunable, yielding a flexibility to include carbon intensity signal into one of the optimization weights. This might be determined as locating the task closer to regions that rely more on renewables power even if those are slightly higher in their network latency regardless of its carbon intensity. This scheduling variant based on individual carbon-awareness would require only minor changes to the model that was covered in the previous sections and will have a significant effect on how we can achieve to reduce the environmental impact of services deployed across many devices.

That being said, the method is not without its detractors. Demands of the federated learning layer are reasonable stability in connectivity between clouds involved, such that parameters can be aggregated in a timely manner. In other environments, where inter-cloud links are particularly unreliable or where privacy constraints prevent even encrypted interchange of gradients, our approach may be less beneficial due to the predictive guidance. However, the quantum-inspired part is still heavier than the simplest heuristics even if computationally tractable for the scale of experiments here, which could be a limiting, factor in ultra-low-power edge micro-clouds. These points illustrate that the deployment context will be crucial: QI-AMHML is a solution more suitable for high-capacity, interconnected multi-cloud federations rather than very limited resource micro-clusters.

Another thing to consider is how faithful the simulation phase is. Although this research took pains to align MultiCloudSynth-2025 with real traces and validate behaviors in shadow deployments, no simulation can perfectly emulate the emergent properties of live production workloads. In the wild, there still remains a risk of mediocre performance to be nudged into less favorable trajectories naïvely by unmodeled network phenomena or rare fault modes. One possible mitigation would be to run a small exploration thread in production at all times — essentially a low-overhead version of the quantum amplitude perturbations — continually testing previously unseen conditions from which we can feed back to our federated learning pool.

This project has a kind of a broader take away which is optimization and predicting are not necessarily two stage processes. Prediction contours the search in QI-AMHML and search paths reshape predicted. More generally, this symmetry is what allows the method to gracefully adapt to changing load and fault conditions, so these gains weff-clustered see are not just particular to wß-clustered, but unlikely to solely vanish in another form of load. Operators of multi-cloud infrastructure that need to satisfy all three dimensions (performance, energy and resilience) simultaneously, should examine with care these co-evolutionary frameworks as they may redefine the minimum bar for intuition in any state scheduling solution.

Scheduling Overhead and Latency Analysis.

To assess the practicality of the proposed QI-AMHML framework in latency-sensitive environments, we explicitly analyzed the scheduling overhead incurred by the RQWOA-based scheduler and compared it against the actual task execution time. Scheduling overhead is defined as the wall-clock time required to generate an optimized task-to-resource mapping for a given scheduling window, while execution latency refers to the runtime of the scheduled workload on the selected resources.

Experiments were conducted across varying scheduling window sizes and task arrival rates. For lightweight tasks with an average execution time of approximately 200 ms, the proposed scheduler required between 18 ms and 41 ms to converge to a near-optimal solution, depending on the population size and iteration budget. For medium- and large-scale workloads with execution times exceeding 500 ms, the scheduling overhead remained below 7.5% of the total task runtime. These results demonstrate that the optimization process does not dominate the execution pipeline.

Furthermore, the RQWOA optimizer is executed asynchronously with respect to task execution, allowing previously computed schedules to be reused when workload variation is minimal. This design ensures that scheduling latency does not introduce bottlenecks even under dynamic workload conditions. Compared to classical WOA, the proposed method converges faster due to quantum-inspired position updates and adaptive exploration–exploitation balancing, resulting in lower scheduling delay for equivalent solution quality.

Overall, the scheduling overhead introduced by QI-AMHML is sufficiently low to support latency-aware cloud and edge workloads, validating its applicability to real-time and near-real-time scheduling scenarios.

## Conclusion and future work

Herein, this research have introduced the Quantum-Inspired Adaptive Meta-Heuristic—Machine Learning (QI-AMHML): A new resilient and energy-efficient task scheduling approach for heterogeneous multi-cloud ecosystems The dyanmic balance between exploration and exploitation given realtime workload conditions are achieved by incorporating a Reinforced Quantum Whale Optimization Algorithm (RQWOA) along with a federated gradient boosting predictor in an end-to-end feedback loop. Its design makes resilience a first-class concern implemented directly in the scheduler, and in doing so proactively hides fault-resolution latency along with other reducing energy overheads.

Experimental results on large-scale simulation using the MultiCloudSynth-2025 dataset and live shadow deployments over Google Cloud, Microsoft Azure, and Alibaba Cloud show significant improvements over conventional meta-heuristic, ML-only and hybrid baselines. Any clustered multi-fault event would push system to its limits, yet resilience scores were now near SLA-defined maxima, on average improving energy consumption by up to 38.2%, reducing average service delay by 33.5%. Crucially, these benefits were realised without disrupting other important tail-latency distributions — an especially valuable trait in latency-sensitive SLA-driven applications.

More than the quantitative advances, however, what sets QI-AMHML apart is its emergent ability to anticipate: the scheduler is willing to introduce some slight short-term inefficiency in order to better cope with future load shifts or failure modes. Similar to modern green computing’s value-system emphasizing sustainability and stability over operational pragmatism funneling towards throughput driving, this aspect derived from the fortified quantum amplitude algorithm and entropy-weighted predictive guidance.

Right now, though, it has some limits to its use. Federated learning requires some minimal level of inter-cloud coordination and network stability, and the computational footprint of the quantum-inspired update mechanism may be too large for ultra-low-power micro-cloud or edge deployment. These limitations also represent a potential future area for further refinement. Lastly, though the synthetic-real hybrid dataset used here encompasses a wide range of workloads, additional tests in even less predictable public workloads — particularly ones that exhibit seasonal or market-driven usage spikes — will provide further confirmation on whether the behaviors are generalizable.

The next steps will build along three major axes. That first entails designing a carbon-constrained scheduling mode where some of the empirical optimization criteria now include actual real-time values for regional carbon intensity, which could dictate how the framework moves workloads to more clean power as it operates. Next, This research intend to incorporate spot-market volatility modeling so that the scheduler can also place VMs cost-consciously with: market prices dynamics prediction as a extension of subject assuming more and more suppliers will give options for spot/preemptible pricing. Third, this research see a natural extension in lifting our quantum-inspired piece to a multi-level superposition model that could represent more complex task–resource mappings beyond simple binary presence/absence relationships, we believe this might enhance search resolution particularly in very heterogeneous environments.

Finally, the QI-AMHML framework demonstrates that quantum-inspired search mechanisms and federated predictive models are not just compatible, rather they are synergistic when established together within a real-time co-evolutionary loop. Integrating resilience, sustainability, and adaptability natively into the core elements of scheduling lays down the initial building block for resilient, green and fault-tolerant multi-cloud orchestration systems of next-gen.

## Data Availability

The datasets used and/or analysed during the current study available from the corresponding author on reasonable request.
